# Percentages of oleic acid and arachidonic acid are inversely related in phospholipids of human sera

**DOI:** 10.1186/1476-511X-12-106

**Published:** 2013-07-19

**Authors:** Arne Torbjørn Høstmark, Anna Haug

**Affiliations:** 1 Institute of Health and Society, Section of Preventive Medicine and Epidemiology, University of Oslo, Norway, Box 1130, Blindern 0318, Oslo, Norway; 2 Department of Animal and Aquacultural Sciences, The Norwegian University of Life Sciences, Box 50031432, Ås, Norway

## Abstract

**Background:**

Many health effects of oils rich in oleic acid (18:1 n9) seem to be opposite those of arachidonic acid (20:4 n6), i.e. concerning cardiovascular risk. In recent study in rats we observed that percentages of oleic acid and arachidonic acid were inversely related in total serum lipids. In the present work we investigate whether an inverse relationship between this couple of fatty acids also exists in the phospholipid fraction of human sera.

**Methods:**

The study group consisted of 11 men and 35 women. Mean age was 23.8 ± 2.5 years (mean ± SD), and the body mass index was 23.5 ± 3.2 kg/m^2^. After fasting overnight, blood was drawn and the concentration of fatty acids in serum phospholipids was determined, using gas chromatography. We studied the association between percentages of oleic acid and arachidonic acid using bivariate correlations (Pearson), and multiple linear regressions.

**Results:**

We found an inverse relationship (r = −0.563, p < 0.001; n = 46) between % oleic acid and % arachidonic acid in the serum phosholipid fraction of the 46 fasting subjects. By multiple linear regression, and % 20:4 n6 as the dependent variable, the inverse association with % 18:1 n9 persisted when controlling for sex, age, body mass index, and percentages of the other fatty acids measured (t = −17.6, p < 0.001). Per cent 20:4 n6 seemed to correlate negatively (r = −0.289, p = 0.05) with the (18:1 n9)/(18:0) ratio, estimating Delta9 desaturase activity, and % oleic acid correlated negatively (r = −0.321, p = 0.029) with the (20:4)/(18:2) ratio, estimating desaturases/elongase activities.

**Conclusions:**

In a group of healthy human subjects, percentages of oleic acid and arachidonic acid were inversely related, and the inverse association persisted when controlling for possible confounding variables. The findings might contribute to explain positive health effects of foods rich in oleic acid.

## Background

It is widely accepted that oleic acid (18:1 n9), and oleic acid rich foods such as olive oil may have many beneficial health effects. Among such effects are improved insulin sensitivity, and endothelium-dependent flow-mediated vasodilatation [1], lowering of LDL cholesterol [2,3] and an increase in HDL cholesterol [4]. If lipids in LDL are enriched in oleic acid, the particles will be less liable to be oxidized [5], a property that is of significance for the normal metabolism of LDL [6]. Furthermore, intake of oleic acid seems to be associated with reduced blood pressure [7]. Thus, many of the effects of oleic acid may serve to reduce the risk of cardiovascular diseases. Additionally, the fatty acid may have anticarcinogenic and anti inflammatory effects [8-10].

Although beneficial effects of oils rich in oleic acid have been reported, the mechanisms by which such oils might have beneficial health effects are still incompletely understood. Various antioxidants present in e.g. virgin olive oil, as well as the high content of oleic acid, could partly explain the health effects.

When considering the reported beneficial health effects of oils rich in oleic acid, it occurred to us that many of the positive effects would be anticipated if the fatty acid works to counteract effects of arachidonic acid (20:4 n6). This fatty acid is formed in the body from linoleic acid (18:2 n6), a major constituent in many plant oils, and is converted by cyclooxygenase and lipoxygenase into various eicosanoids, i.e. prostacyclines, thromboxanes and leukotrienes [11]. Arachidonic acid derived thromboxane A_2_ (TXA_2_) and leukotriene B_4_ have strong proinflammatory and prothrombotic properties [12,13]. Furthermore, endocannabinoides, which are derived from arachidonic acid, may have a role in adiposity and inflammation [14].

An interaction between oleic acid and arachidonic acid was suggested several years ago in the rat [15]. More recently, Cicero et al. [5] showed in human subjects that supplementation with a high dose of olive oil for 3 weeks resulted in an increase in LDL oleic acid and a decrease in linoleic and arachidonic acid. Also in chicken breast muscle a negative 18:1 n9 vs. 20:4 n6 association was observed [16].

One mechanism by which oleic acid could counteract those of arachidonic acid is to reduce the relative abundance of arachidonic acid in serum and tissues. Conceivably, increased supply of oleic acid might reduce that of arachidonic acid by pure mass action. This suggestion raises the question of whether an exchange of 20:4 n6 for 18:1 n9 is specific for this couple of fatty acids.

Inverse regulation could also be effected through more specific metabolic feedback regulation. For example, a reduced percentage of arachidonic acid would be expected if oleic acid inhibits Delta-6 desaturase, Elongase-5 (Elovl-5) and/or Delta-5 desaturase, the enzymes governing formation of arachidonic acid from linoleic acid. Conversely, inhibition by arachidonic acid of Delta-9 desaturase should lower percentage oleic acid, and previous studies suggest that this latter mechanism might take place [17].

It seems that the Delta-9 desaturases are of considerable physiological significance. Thus, regulation of the amount of monounsaturated fatty acids (MUFA) has the potential to affect a variety of key physiological variables, such as insulin sensitivity, metabolic rate, adiposity, atherosclerosis, cancer and obesity [17,18].

In accordance with the above considerations, in a rat study we recently reported that percentages of oleic acid and arachidonic acid in total serum lipids were inversely related, a finding which prevailed when controlling for percentages of the other fatty acids measured [19]. This observation raises the question of whether a similar relationship might exist also in human sera. The aim of the present work was to examine this possibility. In the present work we have used the phospholipid fraction of serum, since this fraction is a valid marker for dietary fat intake [20], and is also the precursor of eicosanoids.

## Results

### Characteristics of the group

Mean age of the study group (11 men and 35 women) was 23.8 ± 2.5 (mean ± SD), ranging from 19 to 30 years. The body mass index was 23.5 ± 3.2 kg/m^2^ with a range of 17.5 to 33.2.

### Bivariate association between percentage oleic acid and arachidonic acid in serum phospholipids

**Figure 1:**
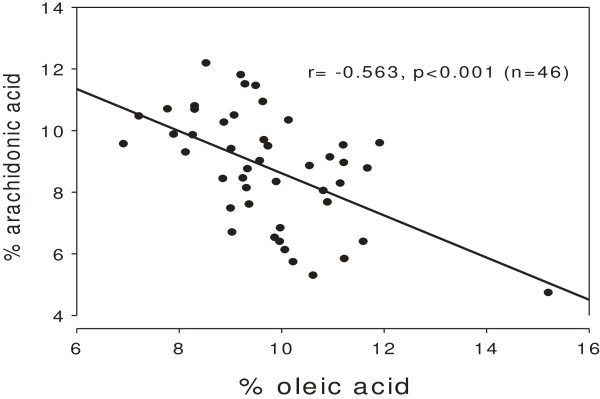
**Association between percentages of oleic acid (18:1 n9) and arachidonic acid (20:4 n6) in serum phospholipids of 46 subjects; p < 0.001 for the negative association.** Note broken axes.

There was an inverse relationship (r = −0.563, p < 0.001; n = 46) between percentages of 18:1 and 20:4 in phospholipids of human serum (Figure 1). There seemed to be an outlier in the scatter plot. Removing this value, we found r = −0.447, p = 0.002 (n = 45).

### Multiple linear regression to evaluate the relationship between oleic acid and arachidonic acid in serum phospholipids, as influenced by other variables

Both the absolute and relative amounts of many fatty acids are intercorrelated, and the inverse relationship between oleic acid and arachidonic acid could possibly be attributed to co-variation with other fatty acids. Conceivably, increase in the percentage of one particular fatty acid must be accompanied by a reduction in the percentage of one or more of the other fatty acids. We accordingly studied the association between % 18:1 n9 (main independent variable) and % 20:4 n6 (dependent variable), using multiple linear regression, and controlling for potential covariates, such as sex, age, BMI, and percentages of the other fatty acids measured. As shown in Table 1, the association between oleic acid and arachidonic acid remained significant when controlling for sex, age, body mass index (Model 2, Table 1), and also when percentages all of the other fatty acids measured were entered simultaneously as independent variables (Model 3). The association between percentages of oleic acid and arachidonic acid seemed to be strengthened in Model 3, as judged from the magnitude of the standardized regression coefficient.

**Table 1:** Association between percentages of arachidonic acid (dependent variable) and oleic acid (independent variable under investigation), as influenced by other variables, multiple linear regression

	**Model**	**B (SE)**	**Beta**	**t**	**p**
1	No adjustment	−0.69 (0.16)	−0.54	−4.2	0.003
2	Model 1 + sex + age + body mass index	- 0.55 (0.17)	−0.43	−3.2	<0.001
3	Model 2 + the other fatty acids measured	- 0.88 (0.05)	−0.69	−17.6	<0.001

Dependent variable = % 20:4 n6. B = unstandardized regression coefficient, Beta = standardized coefficient. Model 1 is without adjustment; Model 2 = Model 1 + adjustment for sex, age, and body mass index (BMI); Model 3 = Model 2 + control for relative amount of all of the other fatty acids measured (see Methods).

**Figure 2:**
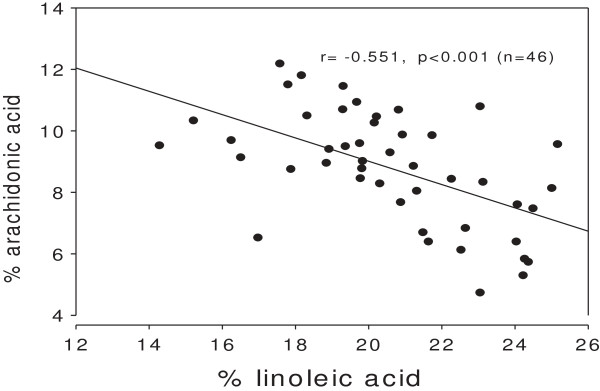
**Association between percentages of linoleic acid (18:2 n6) and arachidonic acid (20:4 n6) in serum phospholipids of 46 subjects; p < 0.001 for the negative association.** Note broken axes.

In accordance with the precursor/product relationship between linoleic acid and arachidonic acid, their relative amounts correlated negatively (Figure 2).

### Is the inverse relationship between percentages of 18:1 n9 and 20:4 n6 related to feedback inhibition of desaturases?

One explanation of the inverse oleic acid vs. arachidonic acid association could be that Delta-5 or 6 desaturases and/or Elongase-5 are inhibited by oleic acid, and/or conversely, that Delta-9 desaturase is inhibited by arachidonic acid. Using product/precursor ratios as crude estimates of desaturase activities, i.e. the oleic acid/stearic acid (18:0) ratio for Delta-9 desaturase, and the arachidonic acid/linoleic acid (18:2 n6) ratio to estimate Delta-5/6 desaturase and Elongase-5, we did find inverse relationships in the study group consisting of 46 subjects (r = −0.289, p = 0.05) for % arachidonic acid vs. the oleic acid/stearic acid ratio; and r = −0.321, p = 0.029 for % 18:1 vs. the arachidonic acid/linoleic acid ratio (results not shown otherwise). However, applying Model 2 and 3 referred to above in multiple linear regression analysis, with the oleic acid/stearic acid ratio (arachidonic acid/linoleic acid ratio) as dependent variables, and per cent arachidonic acid (oleic acid) as the independent under investigation, there was no longer any significant association with the desaturase indexes.

## Discussion

The present work suggests that there is an inverse relationship between percentages of oleic acid and arachidonic acid in serum phospholipids of fasting human subjects. This finding is accordingly in support of our recent study in rats, showing an inverse relationship between this couple of fatty acids in total serum lipids [19].

As judged from the correlation coefficient, the strength of the negative bivariate association was lower in the present human study than in our previous rat trial., This difference might be attributed to differences in the homogeneity of the groups to be compared, i.e. rats and human subjects. We used male rats only, and all of the rats had the same age. They were also in other respects a very homogeneous group, for example concerning genetics and environmental conditions, such as diet, temperature, day/night cycle and general housing. Unlike this, the human subjects included both sexes; the age of the participants was different, and the body mass index varied from one subject to the other. Additionally, there might have been great variation among the subjects in various environmental and lifestyle factors. However, in spite of these many differences, the multiple regression analysis showed that the inverse relationship between percentages of oleic acid and arachidonic acid prevailed when controlling for sex, age, body mass index, and for other fatty acids.

In the rat, an interaction between oleic acid and linoleic acid, the precursor of arachidonic acid, was reported several years ago [17], and in a multi-center randomized cross-over study involving 200 healthy European subjects, Cicero et al. more recently [5] showed that a 3 weeks supplementation with olive oil resulted in an increase in LDL oleic acid and a decrease in linoleic and arachidonic acid. The increase in oleic acid/linoleic acid ratio was accompanied by reduced levels of isoprostanes, biomarkers of oxidative stress.

The finding of a negative correlation between oleic acid and arachidonic also in chicken breast muscle [16] would be in support of the contention that the inverse relationship between this couple of fatty acids exists in many species. Furthermore, in accordance with this suggestion, Kudo et al. [21] observed in mice treated with perfluorinated fatty acids, zenobiotics used as surfactants in various industrial products, that oleic acid was inversely related to arachidonic acid in hepatic phospholipids and total lipids.

The present study does not provide information to explain how the inverse association is brought about. One possibility is that the associations might have been caused by changes in the relative amount of other fatty acids. Conceivably, increase in the percentage of one particular fatty acid must be accompanied by a reduction in the percentage of one or more of the other fatty acids.

However, the inverse association between 18:1 n9 and 20:4 n6 prevailed with high significance in linear regression models when controlling for many factors, including all of the other fatty acids measured. It would appear, therefore, that the inverse association between these fatty acids is not caused by co-variation with other of the factors studied.

We raise the question of how the inverse association between percentages of the two fatty acids is governed. The present study does not clarify metabolic details to explain the inverse relationship. One possibility is that, by pure mass action, increased supply of oleic acid might replace arachidonic acid in various compartments, such as in the lipids of cell membranes. This type of mechanism was previously suggested by the results of a diet trial in chicken [16]. We do not know, however, whether oleic acid by pure mass action has a particular ability to be exchanged for arachidonic acid, or whether this mechanism goes for other fatty acids as well.

It has been suggested that oleic acid is a weak competitive inhibitor of cyclooxygenases [22], which catalyzes conversion of the C20 PUFAs arachidonic acid and eicosapentaenoic acid into prostaglandins, thromboxanes and leucotrienes [11].

Another mechanism serving to explain the inverse relationship could be that oleic acid acts as an inhibitor of Delta-5/6 desaturases and/or Elongase-5 (Elovl-5) so as to reduce the formation of arachidonic acid. Additionally, the possibility exists that arachidonic acid might inhibit the formation of oleic acid by inhibition of Delta-9 desaturase. Possibly, inhibition by arachidonic acid of Delta-9 desaturase gene transcription might be involved, since previous studies suggest that PUFAs of both the n6 and n3 families can inhibit this transcription [18]. However, although a bivariate relationship appeared to exist in the present human study, the association did not attain statistical significance when controlling for many factors, including the other fatty acids measured. It should also be kept in mind that the desaturase indexes used in this study are only crude estimates of the enzyme activities, and more direct methods are needed to confirm whether the suggested hypotheses are valid.

The present results would seem to be in accordance with a previous report showing an increase in LDL oleic acid and a decrease in LDL arachidonic acid, in response to ingesting 25 ml olive oil per day for 3 weeks [5].

Conceivably, also regulation of fatty acid uptake in the intestines, liver and other tissues, e.g. by various fatty acid transporters could be involved, as well as regulation of the catabolism of the fatty acids. Our study was not designed however to elucidate these questions. Finally, it is tempting to speculate whether genetic factors govern the inverse association between oleic acid and arachidonic acid observed among supposedly similar rats.

### Do the findings have health implications?

It would appear that many of the alleged positive health effects of oleic acid should be expected if oleic acid acts to counteract effects of, or reduce the relative amounts of, arachidonic acid. Possibly, a reduced relative amount of arachidonic acid, associated with a high percentage oleic acid, might reduce the formation of metabolites such as TXA2 or LTB4, but we have no data to substantiate this hypothesis. Whatever the mechanisms might be, the present results support our previous observation that percentages of oleic acid and arachidonic acid are inversely related, but further studies are required to evaluate possible health implications of the finding.

## Conclusions

We have observed a consistent inverse relationship between the relative abundance of oleic acid and arachidonic acid in the phospholipid fraction of fasting human sera, prevailing after controlling for many possible confounding factors. The finding raises the question of whether this relationship is a general one across various species, and whether there might be a metabolic feedback regulation between the two fatty acids. Further studies are required to elucidate to what extent the present finding might contribute to explain health effects of foods rich in oleic acid.

## Methods

### Subjects

Forty-six healthy volunteers, 11 men and 35 women, aged 20 to 29 years participated in the study. Most of the participants were students at the Norwegian University for Life Science. The majority had normal body mass index (BMI); individual BMI ranged from 17.5 to 33.5.

### Ethical approval

The study was conducted according to the guidelines laid down in the Declaration of Helsinki, and all procedures involving human subjects were approved by the Regional Committee for Medical Research Ethics and by the Norwegian Data Inspectorate, reference number 28016 from the Norwegian Social Science Data Service. Written informed consent was obtained from all the subjects, and they were informed that they could quit the study whenever they wanted without giving any reason.

### Blood samples, anthropometric and blood pressure measurements

The participants were weighed, using the same scale (Soehnle Digital personal scale, 467017201, Germany. The height was measured using a wall mounted stadiometer. BMI = weight/height^2^ (kg/m^2^).

Blood samples were collected from an antecubital vein of fasting subjects (minimum 12 h fast), between 07.00 and 10.30 A.M. The blood samples were left for 0.5 – 2 h at room temperature and centrifuged at 1300 g for 12 minutes. The sera were frozen and kept at −20°C until analyzed. Blood sampling and determination of body weight and height were done at the local medical health center.

### Serum lipid analyses

Fatty acids in serum phospholipids were determined using the following method: After thawing the serum samples overnight at 4°C, and vortexing for 5 sec, dichloromethane/methanol was added to 200 μl samples, together with 100 μl internal standard (1,2 diheptadecaonyl-*sn*-glycero-3-phosphatidylcholine). After shaking and centrifugation, the supernatants were transferred to new vials and washed in 0.9% NaCl solution. Lower phases were transferred to SPE columns. Neutral lipids were washed out with dichloromethane/isopropanol and MTBE/formic acid. Phospholipids were eluted with methanol. After evaporation to dryness in a vacuum centrifuge, phospholipids were transmethylated with sodium metoxide, and fatty acid methyl esters (FAME) were extracted by hexane before GC analysis. Analysis was performed on a 7890A GC with a split/split less injector, a 7683B automatic liquid sampler, and flame ionization detection (Agilent Technologies, Palo Alto, CA). Separation was performed on a SP 2380 (30 m × 0.22 mm i.d. × 0.25 μm film thickness) column (Supelco, Inc., Bellefonte, PA).

Determination of serum total cholesterol, HDL cholesterol (HDL), LDL cholesterol (LDL), triacylglycerol (TAG) and CRP was performed at Fürst Medical Laboratory, Norway, using routine laboratory methods (SP 03–05 Avida 2400).

### Estimates of fatty acid desaturases

To estimate Delta-9 desaturase, we used the (18:1 n9)/(18:0) ratio. In the present article we may use the term “Delta-5 desaturase” for the (20:4 n6)/(18:2 n6) ratio, keeping in mind that this ratio also includes activities of Elongase-5 and Delta-6 desaturase.

### Statistical analysis

The relationship between percentages of fatty acids was assessed by correlation (Pearson), and by linear regression, with % arachidonic acid as the dependent variable and % oleic acid as the independent variable under investigation. We made 3 regression models: Model 1 is without adjustment; Model 2 = Model 1 with adjustment for sex, age, and body mass index, and. Model 3 = Model 2 + adjustment for percentages of the other fatty acids measured. Results are presented in tables showing mean values ± SEM, or as scatter plots with the regression line included. SPSS 19.0 was used for the regression analyses and Sigma Plot 2001 for producing the figures. A significance level of 0.05 was accepted.

## Competing interests

The authors declare that they have no competing interests.

## Authors’ contributions

ATH conceived and designed the study, analyzed and interpreted the data, and drafted the article. AH contributed substantially to the interpretation of data and revising it critically for important intellectual content. Both the authors approved the final version to be published.
